# Postoperative adjuvant TACE-associated nomogram for predicting the prognosis of resectable Hepatocellular Carcinoma with portal vein Tumor Thrombus after Liver Resection

**DOI:** 10.7150/ijbs.46896

**Published:** 2020-10-23

**Authors:** Fuchen Liu, Xinggang Guo, Wei Dong, Wenli Zhang, Shuxun Wei, Shutong Zhang, Xiuli Zhu, Weiping Zhou, Jinmin Zhang, Hui Liu

**Affiliations:** 1 The Third Department of Hepatic Surgery, Eastern Hepatobiliary Surgery Hospital, Second Military Medical University, Navy Medical University, Shanghai 200438, China.; 2 Changhai Hospital, Second Military Medical University, Navy Medical University, Shanghai 200438, China.; 3 The First Department of General Surgery, Changzheng Hospital, Second Military Medical University, Navy Medical University, Shanghai 200438, China.; 4 Department of Nephrology, First Affiliated Hospital, Anhui Medical University, Hefei, China.; 5 Department of Gastroenterology, Anhui Provincial Hospital, University of Science and Technology of China, Hefei, 230001, China.; 6 Department of Anesthesiology, Eastern Hepatobiliary Surgery Hospital, the Second Military Medical University, Shanghai, China.

**Keywords:** Postoperative Adjuvant TACE, HCC with PVTT, Hepatectomy, Nomogram, Prognosis

## Abstract

**Background:** To explore the effects of postoperative adjuvant transarterial chemoembolization (PA-TACE) on the prognosis of HCC patients with Portal Vein Tumor Thrombus (PVTT) undergoing resection, and to develop a PA-TACE-related nomogram for predicting survival individually.

**Patients and Methods:** Two hundred and ninety-three consecutive HCC patients with PVTT under R0 hepatectomy were recruited. Forty-seven cases had recurrence within one month after surgery. The remaining 246 cases consisted of 90 PA-TACE and 156 non-PA-TACE cases. COX regression analysis was performed for overall survival (OS) or recurrence-free survival (RFS) of these 246 cases, allowing the derivation of independent factors that were integrated into the nomogram. C-index, calibration curves, and risk stratification were performed to evaluate the performance and discriminative power of the nomograms.

**Results:** In 246 patients without recurrence within one month after surgery, the OS and RFS for the PA-TACE group were significantly better than those for the non-PA-TACE group (P<0.0001, P<0.0001, respectively). After Cox regression analysis of OS or RFS, PA-TACE-related nomogram models were constructed. The C-index of the PA-TACE-related nomogram for OS and RFS was 0.72 and 0.73, respectively. Calibration curves revealed a good agreement between predictions and observations for the nomograms. Based on the nomogram-related risk stratification, Kaplan-Meier curves showed powerful discriminative ability.

**Conclusions:** PA-TACE therapy improved the survival of HCC patients with PVTT undergoing hepatectomy. Accurate nomogram models were developed for predicting the individual survival and recurrence of these patients.

## Introduction

Hepatocellular carcinoma (HCC) is one of the most common malignancies. Once diagnosed, however, most HCC is terminal and has lost the chance for radical resection 1, especially in patients with portal vein tumor thrombus (PVTT) which has a high incidence of 44%-62.2% 2. According to the Barcelona Clinic Liver Cancer (BCLC) guidelines, although sorafenib is the recommended treatment for HCC patients with PVTT 3, transarterial chemoembolization (TACE) and liver resection are still considered to be options 4, 5.

In advanced HCC with PVTT, TACE is commonly recommended for a subset of patients 6. A previous prospective study showed that TACE for HCC patients with PVTT achieved both higher 12- and 24-month overall survival rates than the conservative approach 7. Other reports have also shown that TACE is safe and feasible compared with other conservative treatments for HCC patients with PVTT 8.

Surgical resection and liver transplantation are still regarded as the curative modalities for advanced HCC, including cases with port vein invasion 9. The high costs and shortage of organ donors are significant barriers for liver transplantation 10. Many previous studies have revealed that liver resection for HCC patients with PVTT has a survival benefit compared with interventional treatment and other conservative measures 4, 8, 11. In Japan, one previous study compared 1058 surgical patients with 1058 non-surgical patients with HCC and PVTT using propensity score matching, and this study concluded that the surgical approach should be applied for selected HCC patients with tumor invasion limited to the first-order branch of the portal vein, as these patients obtained a longer survival outcome than those receiving non-surgical treatment 12. However, the significant incidence of postoperative recurrence and metastasis was found to be a critical challenge for surgical therapy in HCC with PVTT 13, 14.

Nowadays, treatment strategies for HCC with PVTT remain controversial 15. Various combination therapies, including TACE, radiotherapy, and sorafenib, are commonly used for HCC patients with PVTT, especially TACE treatment 15, 16. Our previous study developed a novel and stable nomogram model for precise individualized therapy for hepatitis B virus (HBV)-related HCC with postoperative adjuvant TACE (PA-TACE). Notably, we have not seen the individual prediction model associated with PA-TACE in HCC patients with PVTT.

In this present study, we explored the influence of PA-TACE on the survival and recurrence of patients with HCC and PVTT who had undergone resection. PA-TACE was usually performed one month after the surgical resection. We aimed to construct a PA-TACE-related nomogram model for predicting the survival risk probability of these advanced HCC patients with port vein invasion who had undergone liver surgery.

## Methods

### Patients

HCC patients with type I/II PVTT, diagnosed according to Cheng's PVTT classification 17, who had undergone R0 radical liver resection in the Eastern Hepatobiliary Surgery Hospital (EHBH) from January 2008 to May 2013 were considered for inclusion. In the retrospective study, candidate patients were recruited based on the following inclusion criteria: (1) diagnosed with HCC pathologically; (2) no preoperative treatment; (3) Child-Pugh A and B; (4) type I/II PVTT; (5) initially underwent R0 surgical resection; (6) treated with PA-TACE one month after resection without other adjuvant treatment. The exclusion criteria were: (1) extrahepatic metastasis or lymph node metastasis; (2) incomplete clinical or follow-up data; (3) diagnosed with other malignancies. It should be noted that PVTT in HCC patients was diagnosed based on typical preoperative radiological indications (computed tomography (CT), magnetic resonance imaging (MRI)) and/or intraoperative and postoperative pathological reporting.

According to the inclusion and exclusion criteria, a total of 293 HCC patients with PVTT were enrolled in the present study. Forty-seven cases recurred within one month after liver resection, 90 cases were treated with PA-TACE one month after liver resection, and 156 cases did not receive any antitumor treatment until relapse one month after hepatectomy.

### Preoperative management and liver resection

After admission, the patients underwent routine abdominal ultrasonography, chest radiography, electrocardiogram, and a pulmonary function test. Additional laboratory tests, including liver function tests, HBV or HCV-related tests, and assay for tumor markers, were also conducted. For patients with large tumors, the remnant liver volume was assessed by a three-dimensional imaging method to prevent postoperative liver failure, according to differences in their underlying liver disease.

After the preoperative safety assessment, patients who had sufficient remnant liver volume and a chance for R0 liver resection were considered candidates for hepatectomy. Anatomical or non-anatomical resection was performed depending on the location, extension, and size of the tumors. A clamp-crushing approach was used for separating the liver parenchyma. Hilar clamping was performed for hepatic portal occlusion depending on the intraoperative situation. R0 liver resection was performed based on an absence of residual tumor tissue and a negative microscopic surgical margin.

### Postoperative management and Adjuvant TACE

Patients who had experienced no recurrence one month after resection and whose liver function had returned to normal underwent postoperative adjuvant TACE. The recurrence criteria are detailed in our previous study 18. Using the Seldinger method, adjuvant TACE was conducted for the entire remnant liver of these postoperative patients, via the proper location of the femoral artery, under the guidance of hepatic and CT angiography. Chemotherapeutic agents, including fluorouracil, epirubicin, and platinum, and embolic agents, including lipiodol and gelatin sponge, were then placed into the proper hepatic artery through the femoral artery using a catheter. The dosage of lipiodol and doxorubicin was evaluated and determined by the body surface area and the remaining liver volume.

### Definitions

Tumor rupture was diagnosed based on preoperative examinations (abdominal ultrasonography, CT, or MRI) and further confirmed by intraoperative detection. With the cutoff of 2000 IU/mL, HBV-DNA load ≥ 2000 IU/mL was defined as high level, and HBV-DNA load < 2000 IU/mL was defined as low level 19. Four hundred ng/ml was considered as the cutoff of AFP (α-fetoprotein level) between the high and low levels 19. A blood platelet level of 100×10^9^ /L was defined as the cutoff level 20.

Tumor differentiation was evaluated and determined according to the Edmondson-Steiner classification 21. The definitions of microvascular invasion (MVI) and satellite lesions were as detailed in our previous studies 18, 22.

### Follow-up and end-points

Considering the high recurrence rate, the patients were followed up every two weeks within the first month after resection. Serum marker tests, abdominal ultrasonography, CT, or MRI was conducted on the patients. For these patients, recurrence within one month after liver resection was considered the primary end-point. When recurrence occurred within one month after surgery, these patients underwent the other treatment modalities.

One month after surgery, a subset of the remaining patients without recurrence underwent PA-TACE treatment. In the first year after liver resection, the patients were followed up every two months, by the routine examinations (serum tumor markers, abdominal ultrasonography, CT or MRI). The secondary end-points were overall survival (OS) (from the date of surgery to patient death or last follow-up date), and recurrence-free survival (RFS), which was calculated from the date of the surgery to the date of first documented tumor recurrence or death after liver resection. And every 6 months was conducted for them subsequently, all patients were followed up until October 2016.

### Statistical analysis

The baseline features of the patients were displayed as mean value (standard error, SD) for continuous variables and frequencies (percentage, %) for categorical variables. The χ^2^ test, or the Fisher exact test, was performed to compare the categorical variables. For continuous variables with normal distribution, the *t*-test was performed, while the Mann-Whitney *U-*test was used for continuous variables with skewed distributions. The Kaplan-Meier method was used to depict the survival curves for OS and RFS, which was compared using the *log-rank* test. Based on the results of the Cox univariate regression analysis, the independent indicators with* P*-values < 0.05 were selected into the next Cox multivariate regression analysis. The above statistical analysis was performed using SPSS software, version 25 (IBM Corp., Armonk, NY).

Independent factors derived from the Cox multivariable analysis were integrated into the nomogram model using the R rms package (*R* version 3.5.1), where the discriminatory ability was evaluated by the C-index. The Kaplan-Meier method was used to depict the calibration curves to assess the agreement between predictions and observations. Risk group stratification (high- and low-risk groups) was based on the total points generated from the nomogram using the *X-title* software 23. A two-tailed *P*-value less than 0.05 was defined as statistically significant.

## Results

### Patients and clinical characteristics

**Figure 1:**
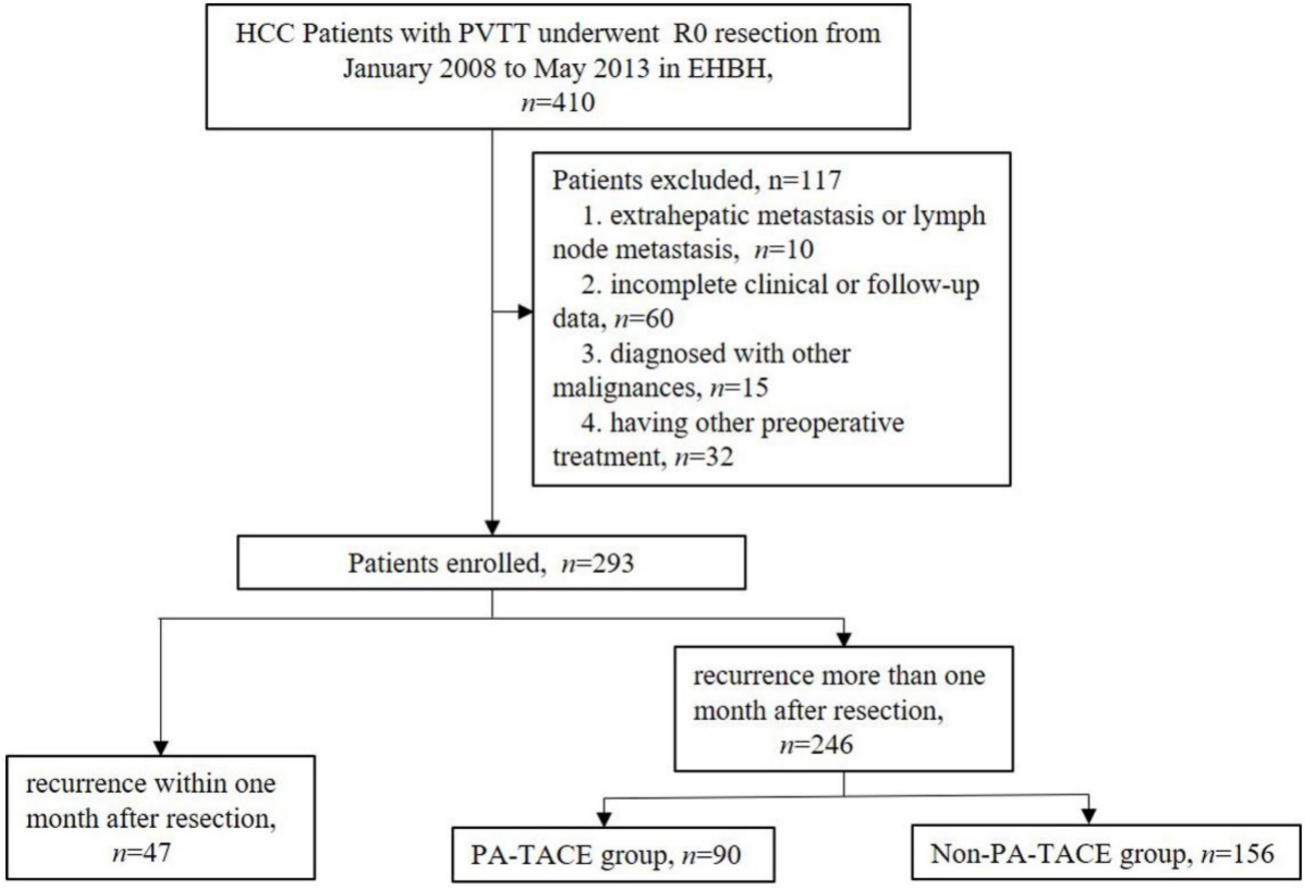
Flow chart showing screening and grouping of HCC patients with PVTT.

A total of 31 clinical features, including 13 preoperative features, 10 intraoperative and pathological factors, and 8 pre-PA-TACE factors, were enrolled into this study, as shown in Supplemental Table 1. Two hundred and ninety-three primary liver cancer patients with PVTT were classified as advanced HCC, according to the BCLC staging; of these, more than 90% had hepatitis B viral infections. A total of 47 cases had a short-term recurrence within one month after surgery (Supplemental Table 1, Fig. 1). The other 246 cases were then divided into two groups (PA-TACE (n=90) vs. non-PA-TACE (n=156)), according to whether they received postoperative adjuvant TACE treatment (Table 1, Fig. 1). Moreover, one to three days before PA-TACE treatment, eight pre-PA-TACE clinical factors were recorded. The same clinical factors in the 156 cases not receiving PA-TACE were also recorded at corresponding times.

### Poor Postoperative RFS rate and OS rate of HCC patients with PVTT after liver resection

After liver resection, the 293 HCC patients with PVTT had a low median overall survival time of 10.05 months, while the 1-, 2-, 3-, 4-, and 5-year overall survival rates were 40%, 21%, 14%, 12%, and 10%, respectively. In terms of cancer recurrence, the 293 cases also had a low recurrence-free time of 6.84 months, with the 1-, 2-, 3-, 4-, and 5-year recurrence-free rates were 12%, 8%, 5%, 5%, and 4%, respectively.

### PA-TACE benefited the survival of HCC patients with PVTT after resection

**Figure 2:**
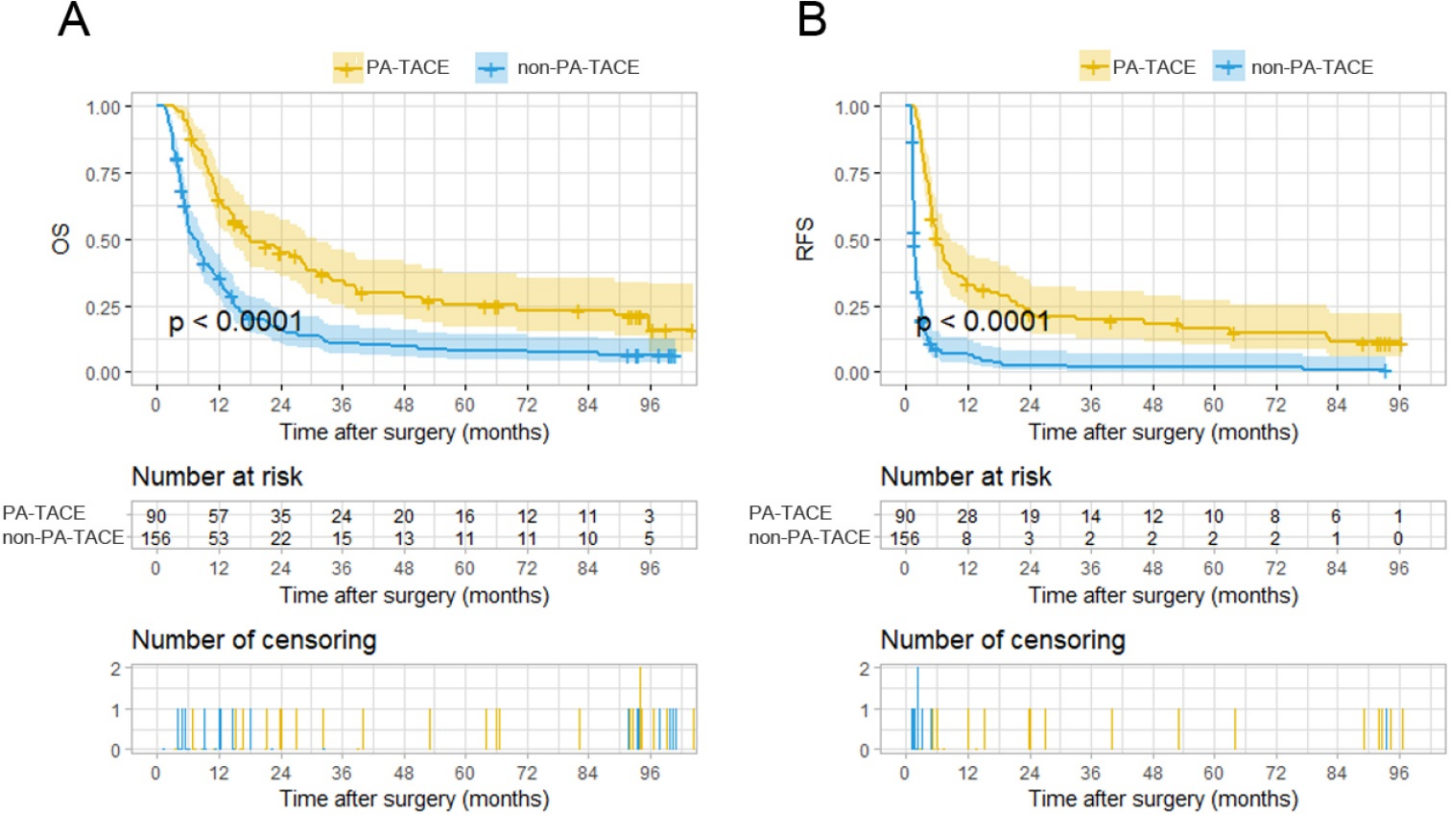
Kaplan-Meier analysis for predicting survival of HCC patients with PVTT with or without postoperative adjuvant TACE. **A.** Kaplan-Meier analysis for OS, **B.** Kaplan-Meier analysis for RFS.

With the exclusion of the 47 cases not suitable for PA-TACE, the two groups, the PA-TACE group (n=90) and the non-PA-TACE group (n=156), comprising the remaining 246 cases had significantly distinct OS and RFS rates, as depicted by KM curves (Fig. 2A-B). The PA-TACE group patients had a median overall survival time of 18.55 months, with 1-, 2-, 3-, 4-, and 5-year overall survival rates of 63%, 39%, 27%, 22%, and 18%, respectively. The non-PA-TACE group patients had a median overall survival time of 9.09 months, with 1-, 2-, 3-, 4-, and 5-year overall survival rates of 34%, 14%, 10%, 8%, and 7%, respectively. At the 1-, 2-, 3-, 4-, and 5-year follow-ups, the overall survival rates for patients of PA-TACE group were significantly higher than those for patients in the non-PA-TACE group (all *P*'s < 0.001).

In terms of tumor recurrence, the PA-TACE group patients had a recurrence-free time of 8.71 months, with 1-, 2-, 3-, 4-, and 5-year recurrence-free rates of 31%, 21%, 16%, 13%, and 11%, respectively. Patients in the non-PA-TACE group had a median overall survival time of 6.32 months, with 1-, 2-, 3-, 4-, and 5-year recurrence-free rates of 5%, 2%, 1%, 1%, and 1%, respectively. At the 1-, 2-, 3-, 4-, and 5-year follow-ups, the recurrence-free rates for patients of PA-TACE group were significantly higher than those for patients in the non-PA-TACE group (*P*<0.001, *P*<0.010, *P*<0.01, *P*<0.001 and *P*<0.001, respectively). These results show that PA-TACE benefited the survival of the patients with HCC and PVTT after resection not only in the OS rate but also in the RFS rate.

According to the Clavien-Dindo classification 24 (Supplemental Table 2), side effects of 90 patients who underwent adjuvant TACE were displayed. Most of the complications of PA-TACE belonged to Clavien-Dindo grade I (86.7%), including fever, abdominal pain, nausea, and vomiting. Two (2.2%) patients experienced grade II complications and needed blood transfusions. Only one patient (1.1%, grade III) underwent abdominal puncture drainage due to massive ascites. No one died from complications of PA-TACE in these patients.

### Independent risk factors for PA-TACE-related RFS and OS of PVTT patients after resection

A total of 90 HCC patients with PVTT underwent PA-TACE therapy one month after hepatectomy and had a better survival rate than the 156 cases without PA-TACE. For PA-TACE related OS, Cox univariate regression analysis revealed that a total of seven risk factors were screened and integrated into further multivariate analysis (Table 2). Further Cox regression analysis identified five clinical features as independent risk factors (Table 3). For PA-TACE related RFS, Cox univariate regression analysis revealed that 10 risk factors were screened and integrated into further multivariate analysis (Table 2), the results of which identified three independent risk factors (Table 3). PA-TACE was also a critical independent risk feature for both OS and RFS of HCC patients with PVTT who had undergone liver resection and PA-TACE. Notably, the HBV DNA load and postoperative antiviral therapy benefited the survival of these groups of patients. Moreover, there is no significant difference between type I and type II PVTT in both OS and RFS of these 90 patients based on the results of Kaplan-Meier analysis (Figure S1).

### Construction of PA-TACE-related RFS or OS Nomogram in Predicting Survival

**Figure 3:**
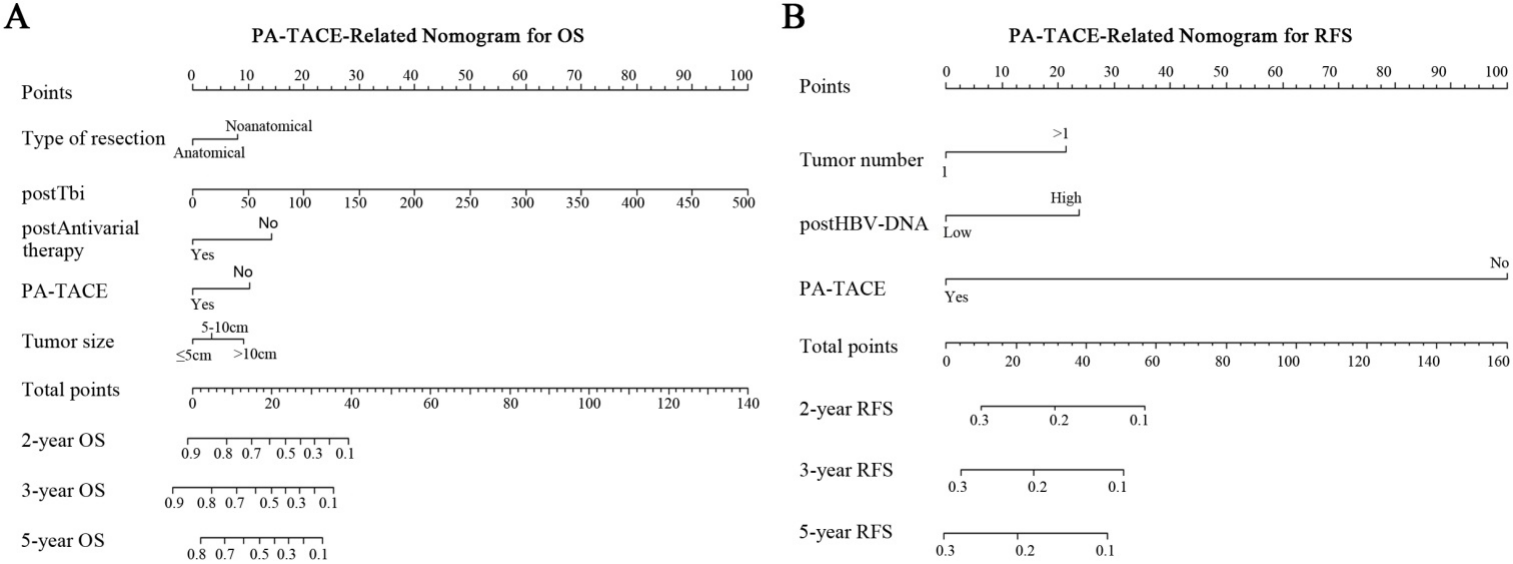
Nomograms for survival of HCC patients with PVTT after liver resection. PA-TACE-related nomogram for OS (**A**) or RFS (**B**).

According to the multivariate analysis of PA-TACE-related OS in the 246 cases, five independent risk factors were integrated into the PA-TACE-related nomogram model: type of resection, tumor size, post-total bilirubin (Tbi), post-antiviral therapy, and PA-TACE. After undergoing resection and PA-TACE, patients who had anatomical hepatectomy or postoperative antiviral treatment had higher OS rates. However, larger tumor size, higher Tbi level, or no PA-TACE were related to a lower OS rate (Fig. 3A).

In the PA-TACE-related RFS nomogram model of 246 cases, three independent risk factors were retained: tumor number, post-HBV-DNA, and PA-TACE. Three indicators, including presence of multiple tumors, and higher postoperative HBV-DNA load or without PA-TACE, were associated with a poorer prognosis of RFS (Fig. 3B).

### The performance of PA-TACE-related Nomograms in Predicting Survival

**Figure 4:**
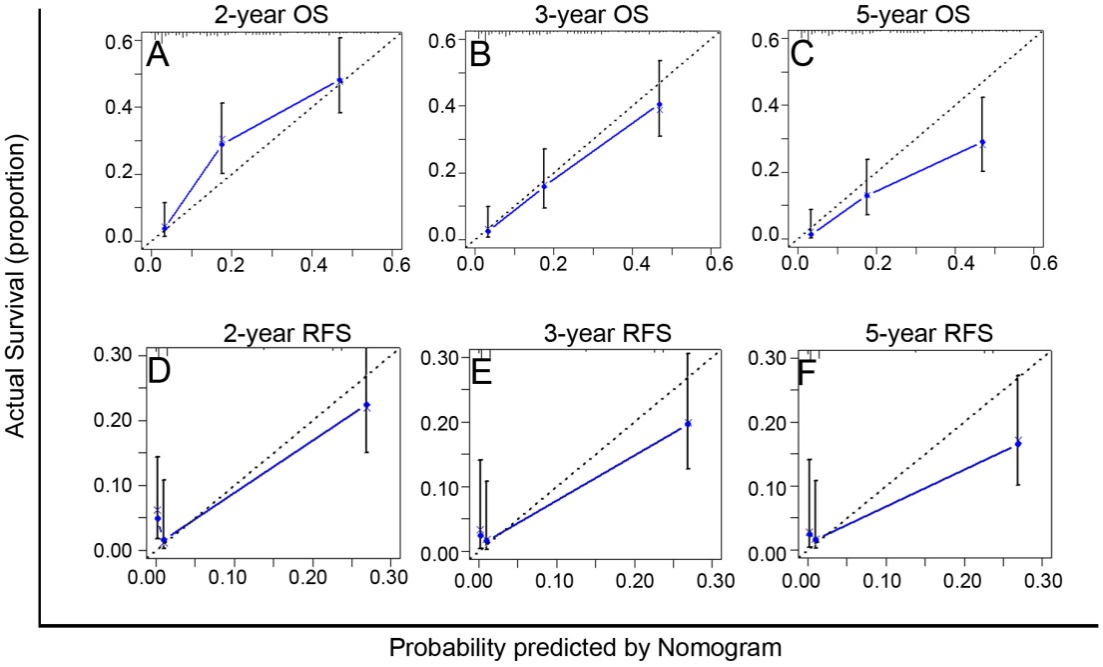
Calibration curves of the PA-TACE-related nomogram models for predicting survival in 246 patients with recurrence more than one month after surgical resection. **A-C.** The calibration curves of the PA-TACE-related nomogram model for predicting OS at 2 years (A), 3 years (B) and 5 years (C); **D-F.** The calibration curves of the PA-TACE-related nomogram model for predicting RFS at 2 years (D), 3 years (E) and 5 years (F). X-axis represents nomogram-predicted probability of survival; Y-axis represents actual survival.

The C-index of the PA-TACE-related OS and RFS nomograms showed good discriminative ability, with values of 0.72 (95% CI: 0.70-0.75) and 0.73 (95% CI: 0.70-0.76), respectively. Furthermore, the calibration curves demonstrated good agreement between the predictions and the observations of 2-, 3-, and 5-year OS (Fig. 4A-C) or RFS (Fig. 4D-F), respectively.

**Figure 5:**
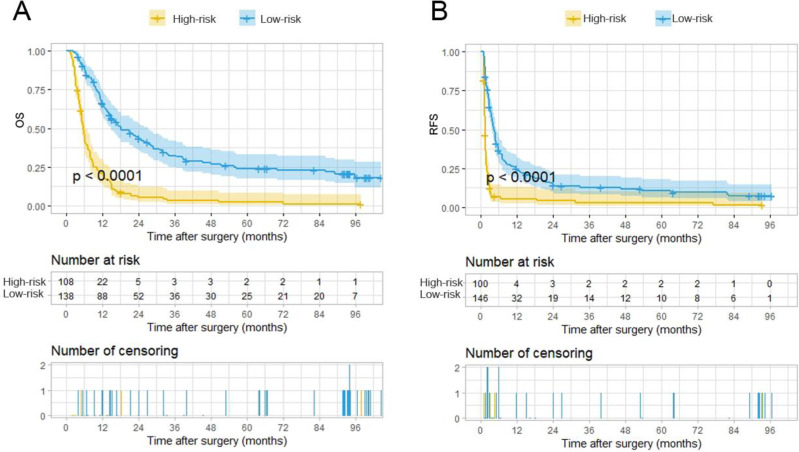
Kaplan-Meier analysis for survival in HCC patients with PVTT with or without PA-TACE according to the stratification risk groups (high-risk and low-risk) derived from PA-TACE-related nomogram models. **A.** Kaplan-Meier analysis for OS, **B.** Kaplan-Meier analysis for RFS.

The total points of the 246 patients ranged between 1.64-133.22 and 0-145.16, according to the PA-TACE-related OS and RFS nomograms. The 246 cases were further classified into a high-risk group and a low-risk group by their corresponding optimal cutoff value for OS or RFS (35.7 for OS, 111.7 for RFS). Based on the risk grouping method, KM curves of OS and RFS were developed, which showed distinct differences between the high- and low-risk groups (*P*<0.0001 for OS,* P*<0.0001 for RFS) (Fig. 5A-B).

## Discussion

Although Sorafenib or lenvatinib are recommended as the first-line therapy for PVTT by many guidelines for advanced HCC treatment 3, 25, 26, recent studies have shown that surgical resection produced better survival outcomes than nonsurgical therapy in HCC patients with PVTT limited to a first-order branch of the main portal vein (MPV) or above 2, 27. Although various novel treatment modalities, including immunotherapy, combination therapy, and interferon, have been introduced in clinics to improve the prognosis of HCC with PVTT after undergoing resection 15, 28, these efforts appear to be insufficient due to high postoperative recurrence and short-term survival time 14, 29. Furthermore, the standard treatment for these HCC patients with PVTT remains controversial. In our study, we showed that PA-TACE improved the prognosis of HCC patients with PVTT who had undergone resection. In addition, we developed PA-TACE-related nomogram models for predicting the survival risk probability of patients with HCC and PVTT.

There has been no effective prediction model for HCC patients with PVTT after resection, especially for PA-TACE-associated cases. Although one study reported a model incorporating PA-TACE for HCC patients with PVTT, it only focused on the outcome of long-term survival time, without exploring the effects of PA-TACE on the recurrence 6. A continuous therapy course, surgical resection and PA-TACE treatment should be considered for HCC patients with PVTT undergoing liver resection. PA-TACE treatment has relatively few complications, although, after adjuvant TACE treatment, HCC patients who had undergone curative liver resection experienced somewhat greater levels of fever, abdominal pain, nausea, and vomiting. We constructed a PA-TACE-related model for postoperative patients suitable for PA-TACE one month after resection, which integrated not only the hepatectomy-associated clinical indicators but also HBV-related factors and liver function indicators before PA-TACE treatment. The PA-TACE-related nomogram models showed good discriminative ability in predicting the prognostic risk probability for these patients. Therefore, the importance of follow-up indicators should be recognized and expanded in HCC patients with PVTT undergoing resection, as these are of great significance for guiding the follow-up and the design of postoperative therapy.

Previous PVTT-related studies only focused on the recurrence and survival differences between PA-TACE and no PA-TACE treatment in HCC patients with PVTT after resection 8. Few reports have paid much attention to the independent risk factors for PA-TACE-related prognosis for these patients. Our study not only demonstrated that PA-TACE could significantly improve the prognosis of HCC with PVTT after surgery, but also analyzed the clinical factors, including preoperative factors, intraoperative and pathological factors, and PA-TACE-related clinical factors one month after liver resection. However, follow-up concerns of postoperative factors have not attracted much interest in previous studies.

The PA-TACE-related model showed that both postoperative antiviral therapy and lower HBV-DNA load benefited the survival of patients with HCC and PVTT who had undergone resection. A previous study from our institution reported that the postoperative antiviral modality reduced the recurrence risk in HCC patients, and improved the long-term survival 30. Therefore, the postoperative HBV-DNA level of HBV-infected patients should be treated with greater caution and given aggressive antiviral treatment. Thus, postoperative antiviral treatment could also apply to the special group of HCC patients with PVTT who undergo resection. Total bilirubin is a critical component of liver failure staging systems 31-33, which also impaired the prognosis of HCC patients 34, 35. Total bilirubin also integrated into the established PA-TACE-related nomogram model for predicting the risk of OS, indicating that liver function should be given sufficient attention and evaluated before PA-TACE for the HCC patients with PVTT who underwent hepatectomy. Bilirubin was also one critical component of the EHBH/PVTT Scoring System for aiding the decision making on surgical resection for HCC patients with PVTT. Moreover, for PA-TACE-related prognosis, both the numbers of tumors and their diameters have been recognized as critical independent risk factors for poor HCC prognosis 22, 36, which was also further confirmed in HCC patients with PVTT by previous studies 13, 36. Our study demonstrated that tumor size reduced the overall survival time and multiple tumors impaired the RFS of these patients.

In terms of the type of resection, the roles of anatomical and non-anatomical hepatectomy approaches in the prognosis of HCC patients are controversial 37, 38. In our study, anatomical hepatectomy had a better postoperative long-term survival time for the HCC patients with PVTT compared with the non-anatomical hepatectomy approach, and anatomic hepatectomy should be preferred for these patients.

For resectable HCC patients with PVTT after liver resection, PA-TACE could be a priority selection, especially the patients with non-anatomical resection, multiple tumor number, larger tumor size, higher post-Tbi level or higher post-HBV-DNA load. However, this study has two limitations. Firstly, the results of our study need to be validated by multicenter cohorts, and prospective studies are also needed. Secondly, due to the higher sensitivity of digital subtraction angiography (DSA) compared to CT or MRI in the detection of tumor lesions, recurrence could not be detected earlier in the non-PA-TACE group patients one month after resection, resulting in a poorer prognosis than the patients with adjuvant TACE.

Moreover, two recent studies seemed to achieve better overall survival in unresectable HCC treated with lenvatinib plus pembrolizumab^39^ (median overall survival, 22 months), or atezolizumab plus bevacizumab^40^ (overall survival at 12 months, 67.2%), compared with the prognosis of PA-TACE group patients after resection in our study (median overall survival, 18.55 months and overall survival at 12 months, 63%). Although the above results should be further explored via future rigorous randomized controlled study, these studies revealed that the targeted and immunological therapy, or their combination with PA-TACE have shown strong vitality and development potential for advanced HCC treatment.

In conclusion, PA-TACE benefited the survival of HCC patients with PVTT undergoing liver resection. Nomogram models were developed and showed good discriminative ability and accuracy for predicting the sequential prognostic risk probabilities. Postoperative antiviral treatment is essential for HBV-infected patients, who should be given more frequent follow-ups and further aggressive therapy.

## Supplementary Material

Supplementary figures and tables.

## Figures and Tables

**Table 1:** Basal clinicopathological characteristics of 246 HCC patients with PVTT with and without PA-TACE

Features	Recurrence more than one month (n=246)			
	Total patients (n=246)	Non-PA-TACE (n=156)	PA-TACE (n=90)	*P*
**Preoperative Factors**				
Age (mean (SD))	48.62 (10.43)	47.88 (10.27)	49.89 (10.62)	0.147
Sex (Male/Female) (%)	226 (91.9)/20 (8.1)	140 (89.7)/16 (10.3)	86 (95.6)/4 (4.4)	0.172
Tumor rupture (Yes/No) (%)	8 (3.3)/238 (96.7)	6 (3.8)/150 (96.2)	2 (2.2)/88 (97.8)	0.750
Child-pugh (A/B) (%)	209 (85.0)/37 (15.0)	141 (90.4)/15 (9.6)	68 (75.6)/22 (24.4)	**0.003**
HBsAg (Positive/Negative) (%)	222 (90.2)/24 (9.8)	144 (92.3)/12 (7.7)	78 (86.7)/12 (13.3)	0.225
HBV-DNA (≥2000/<2000 IU/mL) (%)	128 (52.0)/118 (48.0)	84 (53.8)/72 (46.2)	44 (48.9)/46 (51.1)	0.537
Antiviral therapy (Yes/No) (%)	18 (7.3) /228 (92.7)	9 (5.8) /147 (94.2)	9 (10.0) /81 (90.0)	0.330
AFP (˃400/≤400, ng/ml) (%)	167 (67.9)/79 (32.1)	110 (70.5)/46 (29.5)	57 (63.3)/33 (36.7)	0.308
Tbi (mean, μmol/L) (SD)	16.72 (24.89)	18.51 (30.89)	13.61 (5.24)	0.138
PALB (mean, g/L) (SD)	178.88 (56.10)	184.29 (60.89)	169.51 (45.46)	**0.046**
Alb (mean, g/L) (SD)	41.28 (3.75)	41.22 (3.88)	41.41 (3.54)	0.703
PLT (≥100/<100*10^9^) (%)	29 (11.8)/217 (88.2)	24 (15.4)/132 (84.6)	5 (5.6)/85 (94.4)	**0.036**
INR (mean (SD))	1.01 (0.08)	1.01 (0.08)	1.00 (0.07)	0.482
**Intraoperative and Pathological Factors**				
Transfusion (Yes/No) (%)	92 (37.4)/154 (62.6)	71 (45.5)/85 (54.5)	21 (23.3)/69 (76.7)	**0.001**
Type of resection (anatomical/nonanatomical) (%)	75 (30.5)/171 (69.5)	45 (28.8)/111 (71.2)	30 (33.3)/60 (66.7)	0.553
Hilar clamping time (mean, minutes) (SD)	20.64 (11.98)	20.43 (13.27)	21.00 (9.41)	0.72
Tumor size (<10/5-10/≤5cm) (%)	92 (37.4)/111 (45.1)/43 (17.5)	67 (42.9)/67 (42.9)/22 (14.1)	25 (27.8)/44 (48.9)/21 (23.3)	**0.034**
Tumor Number (>1/1) (%)	86 (35.0)/160 (65.0)	55 (35.3)/101 (64.7)	31 (34.4)/59 (65.6)	1.000
Cirrhosis (Yes/No) (%)	153 (62.2)/93 (37.8)	103 (66.0)/53 (34.0)	50 (55.6)/40 (44.4)	0.135
Tumor capsule (Complete/Incomplete/Absent) (%)	13 (5.3)/112 (45.5)/121 (49.2)	9 (5.8))/62 (39.7)/85 (54.5	4 (4.4)/50 (55.6)/36 (40.0)	0.056
Satellite lesions (Yes/No) (%)	32 (13.0)/214 (87.0)	21 (13.5)/135 (86.5)	11 (12.2)/79 (87.8)	0.935
MVI (Yes/No) (%)	216 (87.8)/30 (12.2)	142 (91.0)/14 (9.0)	74 (82.2)/16 (17.8)	0.067
Edmondson-Steiner grade (III-IV/II) (%)	33 (13.4)/213 (86.6)	11 (7.1)/145 (92.9)	22 (24.4)/68 (75.6)	**<0.001**
**Postoperative and prePA-TACE**				
postHBsAg (Positive/Negative) (%)	222 (90.2)/24 (9.8)	144 (92.3)/12 (7.7)	78 (86.7)/12 (12.3)	0.225
postHBV-DNA (≥2000/<2000 IU/mL) (%)	101 (41.1)/145 (58.9)	71 (45.5)/85 (54.5)	30 (34.4)/60 (65.6)	0.083
postAFP (>400/≤400, ng/ml) (%)	93 (37.8)/153 (62.2)	75 (48.1)/81 (51.9)	18 (20.0)/72 (80.0)	**<0.001**
postTbi (mean (SD))	21.31 (38.82)	25.18 (48.18)	14.59 (5.82)	**0.039**
postAlb (mean (SD))	43.53 (35.09)	40.66 (23.67)	48.52 (48.73)	0.091
postPLT (≥100/<100×10^9^) (%)	38 (15.4)/208 (84.6)	25 (16.0)/131 (84.0)	13 (14.4)/77 (85.6)	0.883
postINR (mean (SD))	1.08 (0.10)	1.08 (0.11)	1.07 (0.09)	0.268
postAntiviral therapy (Yes/No) (%)	25 (10.2)/221 (89.8)	13 (8.3)/143 (91.7)	12 (13.3)/78 (86.7)	0.302

Bold values indicate statistical significance (P < 0.05). HCC, hepatocellular carcinoma; PVTT, Portal Vein Tumor Thrombus; PA-TACE, postoperative adjuvant transarterial chemoembolization; AFP, α-fetoprotein; Tbi, total bilirubin; PALB, prealbumin; Alb, albumin; PLT, blood platelet; INR, international normalized ratio; MVI, microvascular invasion.

**Table 2:** Univariate Cox-regression analysis for predicting OS and RFS in 246 HCC patients with PVTT with and without PA-TACE

Factors	OS			RFS		
	HR	95%CI	*P*	HR	95%CI	*P*
**Univariate analysis**						
Age (mean (SD),Years)	1.000	0.99-1.02	0.584	0.990	0.98-1	0.096
Sex (Male/Female)	1.370	0.83-2.25	0.214	1.270	0.79-2.03	0.327
Tumor Rupture (Yes/No)	1.220	0.57-2.61	0.600	1.320	0.65-2.67	0.448
Child-pugh (A/B)	0.800	0.54-1.18	0.254	0.520	0.35-0.76	**0.001**
HBsAg (Positive/Negative)	0.950	0.6-1.49	0.826	1.360	0.88-2.12	0.167
HBV-DNA (≥2000 /<2000 IU/mL)	1.220	0.93-1.61	0.154	1.210	0.93-1.58	0.158
Antiviral therapy (Yes/No)	0.640	0.36-1.14	0.131	0.860	0.52-1.43	0.566
AFP (>400/ ≤400 ng/ml)	1.360	1.01-1.84	0.043	1.340	1.01-1.79	**0.044**
Tbi (mean, μmol/L)	1.000	1-1.01	0.069	1.000	1-1.01	0.272
PALB (mean, g/L)	1.000	1-1.00	0.634	1.000	1-1.00	0.211
Alb (mean, g/L)	1.000	0.96-1.04	0.926	0.970	0.93-1	0.068
PLT (≥100/<100×10^9^)	1.100	0.73-1.68	0.649	1.320	0.89-1.97	0.168
INR (mean)	3.040	0.49-18.83	0.231	3.140	0.51-19.35	0.217
Transfusion (Yes/No)	1.410	1.07-1.87	0.016	1.270	0.97-1.68	0.088
Type of resection (anatomical/nonanatomical)	0.590	0.43-0.8	0.001	0.870	0.65-1.16	0.328
Hilar clamping time (mean, minutes)	0.990	0.98-1	0.076	1.000	0.99-1.02	0.557
**Tumor size, ≤5 cm**	1.00 (Reference)			1.00 (Reference)		
5 cm-10 cm	1.550	1.01-2.37	0.044	1.360	0.93-1.99	0.111
>5 cm	2.290	1.49-3.51	<0.001	1.760	1.19-2.61	**0.004**
Tumor Number (>1/1)	1.110	0.83-1.47	0.495	1.330	1.01-1.75	**0.042**
Cirrhosis (Yes/No)	1.090	0.82-1.44	0.567	1.110	0.85-1.46	0.449
**Tumor capsule, Complete**	1.00 (Reference)			1.00 (Reference)		
Incomplete	0.840	0.63-1.11	0.223	0.670	0.5-0.88	**0.004**
Absent	1.330	0.72-2.49	0.364	0.930	0.51-1.7	0.081
Satellite lesions (Yes/No)	1.340	0.9-1.98	0.148	1.360	0.93-1.99	0.113
MVI (Yes/No)	1.810	1.15-2.85	0.011	1.940	1.24-3.03	**0.004**
Edmondson-Steiner grade (III-IV/II)	1.150	0.77-1.71	0.501	2.080	1.38-3.14	**<0.001**
postHBsAg (Positive/Negative)	0.950	0.6-1.49	0.826	1.360	0.88-2.12	0.167
postHBV-DNA(≥2000 /<2000 IU/mL)	1.200	0.91-1.58	0.207	1.410	1.08-1.84	**0.012**
postAFP (>400/≤400 ng/ml)	1.720	1.3-2.27	<0.001	1.540	1.17-2.02	**0.002**
postTbi (mean, μmol/L)	1.020	1.01-1.02	<0.001	1.000	1-1.01	0.243
postAlb (mean, g/L)	1.000	1-1.01	0.114	1.000	1-1.00	0.718
postPLT (≥100/<100×10^9^)	0.950	0.65-1.39	0.796	1.280	0.89-1.83	0.179
postINR (mean)	3.330	0.9-12.31	0.071	1.260	0.36-4.39	0.717
postAntiviral therapy (Yes/No)	0.330	0.19-0.58	<0.001	0.890	0.58-1.38	0.609
PA-TACE (No/Yes)	2.240	1.67-3.02	<0.001	3.960	2.94-5.32	**<0.001**

Bold values indicate statistical significance (P < 0.05). OS, overall survival; RFS, recurrence-free survival. HCC, hepatocellular carcinoma; PVTT, Portal Vein Tumor Thrombus; AFP, α-fetoprotein; Tbi, total bilirubin; PALB, prealbumin; Alb, albumin; PLT, blood platelet; INR, international normalized ratio; MVI, microvascular invasion.

**Table 3:** Multivariate Cox-regression analysis for predicting OS and RFS in 246 HCC patients with PVTT with and without PA-TACE

Factors	OS			RFS		
	HR	95%CI	*P*	HR	95%CI	*P*
**Multivariate analysis**						
Type of resection (anatomical/nonanatomical)	0.550	0.4-0.76	**<0.001**			
**Tumor size, ≤5 cm**	1.00 (Reference)					
5-10 cm	1.260	0.81-1.95	0.299			
>10 cm	1.910	1.22-2.99	**0.005**			
**Tumor Number (>1/1)**				1.430	1.05-1.94	**0.023**
postHBV-DNA (≥2000 /<2000 IU/mL)				1.330	1.01-1.77	**0.045**
postTbi (mean, μmol/L)	1.010	1.01-1.02	**<0.001**			
postAntiviral therapy (Yes/No)	0.340	0.19-0.6	**<0.001**			
PA-TACE (No/Yes)	2.06	1.49-2.85	**<0.001**	3.79	2.74-5.23	**<0.001**

Bold values indicate statistical significance (*P* < 0.05). OS, overall survival; RFS, recurrence-free survival. HCC, hepatocellular carcinoma; PVTT, Portal Vein Tumor Thrombus; Tbi, total bilirubin.
