# Supplementary material for: Postoperative adjuvant TACE-associated nomogram for predicting the prognosis of resectable Hepatocellular Carcinoma with portal vein Tumor Thrombus after Liver Resection
Source: Int J Biol Sci. 2020 Oct 23;16(16):3210–20. doi: 10.7150/ijbs.46896 (PMC7645989; doi:10.7150/ijbs.46896)
Supplement: Supplementary file 1 — Supplementary figures and tables. [file ijbsv16p3210s1.pdf]

Supplemental Figure Legends

Supplemental Figure 1. Kaplan-Meier analysis for survival in 90 PA-TACE patients with PVTT after liver resection. A. Kaplan-Meier analysis for OS, **B.** Kaplan-Meier analysis for RFS.

Supplemental Table Legends

Supplemental Table 1. Basal clinicopathologic characteristics of 293 HCC patients with PVTT after liver resection.

Supplemental Table 2. Side effects of 90 patients who underwent adjuvant TACE after liver resection

Supplemental Figure 1

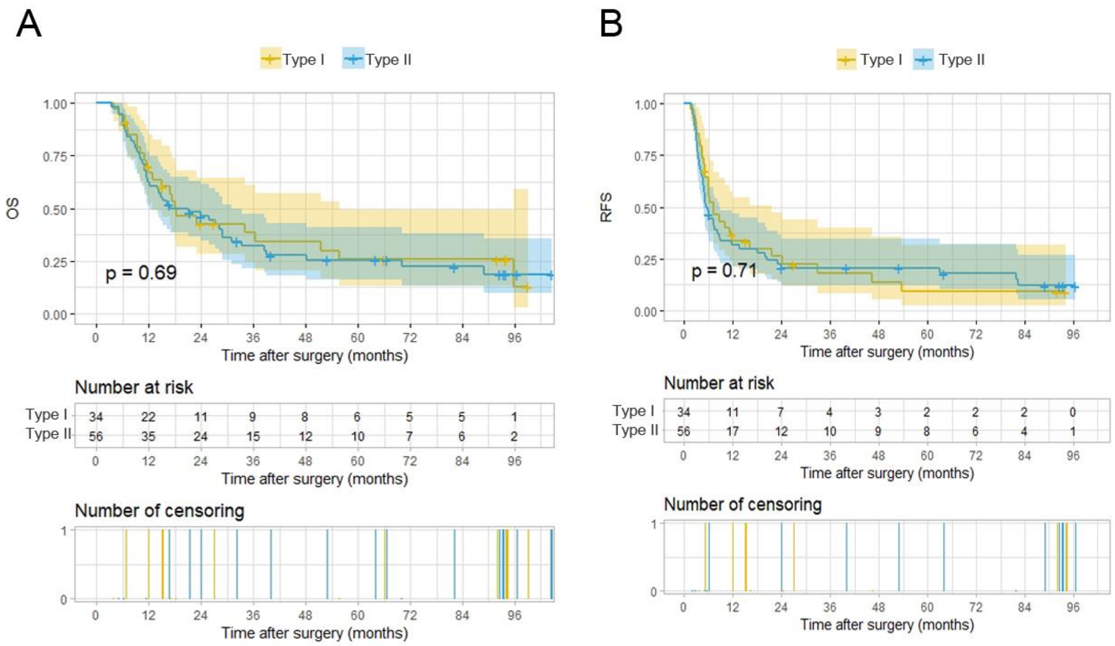

Supplemental Table 1. Basal clinicopathologic characteristics of 293 HCC patients with PVTT after liver resection.

Features	Total patients(n=293)	Recurrence within one month(n=47)	Recurrence more than one month(n=246)	***P***
**Preoperative Factors**				
Age (mean (SD))	48.73(10.77)	49.34(12.55)	48.62 (10.43)	0.674
Sex(Male/Female) (%)	268(91.5)/25(8.5)	42(89.4)/5(10.6)	226(91.9)/20(8.1)	0.780
Tumor rupture(Yes/No) (%)	12(4.1) /281(95.9)	4(8.5)/43(91.5)	8(3.3)/238(96.7)	0.206
Child-pugh(A/B) (%)	242(82.6) /51(17.4)	33(70.2)/14(29.8)	209(85.0)/37(15.0)	**0.026**
HBsAg(Positive/Negative) (%)	269(91.8) /24(8.2)	47(100.0)/0(0.0)	222(90.2)/24(9.8)	0.052
HBV-DNA(≥2000 /<2000 IU/mL) (%)	153(52.2) /140(47.8)	25(53.2)/22(46.8)	128(52.0)/118(48.0)	1.000
Antiviral therapy(Yes/No) (%)	26(8.9) /267(91.1)	8(17.0) /39(83.0)	18 (7.3) /228(92.7)	0.062
AFP(>400/≤400, ng/ml) (%)	202(68.9) /91(31.1)	35(74.5)/12(25.5)	167(67.9)/79(32.1)	0.471
Tbi (mean, μmol/L)(SD)	16.48(22.91)	15.21(5.57)	16.72(24.89)	0.681
PALB(mean, g/L) (SD)	179.65(55.11)	183.66(49.97)	178.88(56.10)	0.587
Alb(mean, g/L)(SD)	41.13(3.81)	40.30(4.05)	41.28(3.75)	0.103
PLT(≥100/<100*10^9) (%)	35(11.9) /258(88.1)	6(12.8)/41(87.2)	29(11.8)/217(88.2)	1.000
INR (mean (SD))	1.01(0.08)	1.03(0.07)	1.01(0.08)	**0.030**
**Intraoperative and Pathological Factors**				
Transfusion(Yes/No) (%)	115(39.2) /178(60.8)	23(48.9)/24(51.1)	92(37.4)/154(62.6)	0.186
Type of resection (anatomical/nonanatomical)(%)	82(28.0)/211(72.0)	7(14.9)/40(85.1)	75(30.5)/171(69.5)	**0.045**
Hilar clamping time (mean, minutes) (SD)	21.04 (12.03)	23.13(12.22)	20.64(11.98)	0.194
Tumor size(>10/5-10/≤5cm) (%)	111(37.9) /135(46.1) /47(16.0)	19(40.4)/24(51.1)/4(8.5)	92(37.4)/111(45.1)/43(17.5)	0.305
Tumor Number (>1/1)(%)	104(35.5)/189(64.5)	18(38.3)/29(61.7)	86(35.0)/160(65.0)	0.786
Cirrhosis(Yes/No) (%)	191(65.2) /102(34.8)	38(80.9)/9(19.1)	153(62.2)/93(37.8)	**0.022**
Tumor capsule (Complete/Incomplete/Absent) (%)	13(4.4) /128(43.7) /152(51.9)	0(0.0)/16(34.0)/31(66.0)	13(5.3)/112(45.5)/121(49.2)	0.055
Satellite lesions (Yes/No) (%)	41(14.0) /252(86.0)	9(19.1)/38(80.9)	32(13.0)/214(87.0)	0.378
MVI(Yes/No) (%)	260(88.7) /33(11.3)	44(93.6)/3(6.4)	216(87.8)/30(12.2)	0.366
Edmondson-Steiner grade(III-IV/II) (%)	45(15.4) /248(84.6)	12(25.5)/35(74.5)	33(13.4)/213(86.6)	0.059

Bold values indicate statistical significance ($P < 0.05$). HCC, hepatocellular carcinoma; PVTT, Portal Vein Tumor Thrombus; AFP, α-fetoprotein; Tbi, total bilirubin; PALB, prealbumin; Alb, albumin; PLT, blood platelet; INR, international normalized ratio; MVI, microvascular invasion.

Supplemental Table 2. Side effects of 90 patients who underwent adjuvant TACE after liver resection

Clavien-dindo grade	No.(%)
**I**	78(86.7)
**II**	2(2.2)
**III**	1(1.1)
**IV**	0(0)
**V**	0(0)
**Total**	81(90)
